# Potential involvement of the extracranial venous system in central nervous system disorders and aging

**DOI:** 10.1186/1741-7015-11-260

**Published:** 2013-12-17

**Authors:** Robert Zivadinov, Chih-Ping Chung

**Affiliations:** 1 Buffalo Neuroimaging Analysis Center, Department of Neurology, School of Medicine and Biomedical Sciences, University at Buffalo, State University of New York, Buffalo, NY, USA; 2 MR Imaging Clinical Translational Research Center, School of Medicine and Biomedical Sciences, University at Buffalo, State University of New York, 100 High St., Buffalo, NY 14203, USA; 3 Department of Neurology, Taipei Veterans General Hospital, Taipei, Taiwan; 4 Department of Neurology, National Yang Ming University of Medicine, Taipei, Taiwan

**Keywords:** Jugular vein reflux, CCSVI, Anatomy, Etiology, Pathophysiology, Classification, Diagnosis, CNS disorders, Aging, Multiple sclerosis, Compensatory mechanism

## Abstract

**Background:**

The role of the extracranial venous system in the pathology of central nervous system (CNS) disorders and aging is largely unknown. It is acknowledged that the development of the venous system is subject to many variations and that these variations do not necessarily represent pathological findings. The idea has been changing with regards to the extracranial venous system.

**Discussion:**

A range of extracranial venous abnormalities have recently been reported, which could be classified as structural/morphological, hemodynamic/functional and those determined only by the composite criteria and use of multimodal imaging. The presence of these abnormalities usually disrupts normal blood flow and is associated with the development of prominent collateral circulation. The etiology of these abnormalities may be related to embryologic developmental arrest, aging or other comorbidities. Several CNS disorders have been linked to the presence and severity of jugular venous reflux. Another composite criteria-based vascular condition named chronic cerebrospinal venous insufficiency (CCSVI) was recently introduced. CCSVI is characterized by abnormalities of the main extracranial cerebrospinal venous outflow routes that may interfere with normal venous outflow.

**Summary:**

Additional research is needed to better define the role of the extracranial venous system in relation to CNS disorders and aging. The use of endovascular treatment for the correction of these extracranial venous abnormalities should be discouraged, until potential benefit is demonstrated in properly-designed, blinded, randomized and controlled clinical trials.

Please see related editorial: http://www.biomedcentral.com/1741-7015/11/259.

## Background

Mounting evidence suggests that a number of inflammatory and neurodegenerative central nervous system (CNS) disorders may be related to vascular factors [1]. While the role of arterial supply abnormalities in relation to the pathology of CNS disorders is well-defined, the role of venous drainage impairment, for example, extracranial venous abnormalities, is largely unknown [2-7]. The complexity, inter-individual variability and frequent asymmetry of the extracranial venous system, compared to the peripheral venous and arterial systems make exploration of the link between intracranial and extracranial pathology extremely difficult [2,8]. Moreover, additional factors, including postural change, cardiac function, respiration, frequent change in lumen diameter, hydration status, hypovolemia and the presence of nearby structures, may influence correct assessments of the veins in regards to the presence of structural or hemodynamic extracranial venous abnormalities [2-7].

Compared with the arterial system, the development of the extracranial venous system is subject to many variations. Therefore, in the past, these variations were acknowledged as non-pathological findings [9-12]. A variety of congenital extracranial venous abnormalities/developmental variants has been described [10,11]. However, investigations aimed to define the nature of these venous abnormalities/developmental variants and their clinical significances are lacking [13,14].

Several CNS disorders, such as transient global amnesia, transient monocular blindness, cough headache and primary exertional headache, have been linked to the presence and severity of uni- or bi-lateral jugular venous reflux (JVR) in the last two decades [15-20]. More recently, an intense interest in better understanding the role of the extracranial venous system in the pathophysiology of CNS disorders has been generated by the introduction of a composite criteria-based vascular condition named chronic cerebrospinal venous insufficiency (CCSVI). CCSVI is characterized by abnormalities of the main extracranial cerebrospinal venous outflow routes that interfere with normal venous drainage, as evidenced by Doppler sonography (DS) [21-23]. It was originally hypothesized that CCSVI implies a pathological condition that leads to abnormal venous drainage of the brain parenchyma and increased susceptibility to multiple sclerosis (MS) [22]. While, the condition was originally described in MS patients, it became immediately clear from the independent results of the first controlled studies that patients with other CNS disorders and healthy individuals may also show a high prevalence of this condition [2,24-27]. However, because healthy individuals do not suffer from CNS disorders, its clinical relevance as a nosological entity was immediately questioned [26,28]. Indeed, as more research studies have become available, the very concept of CCSVI, its diagnostic utility and clinical impact for MS have all been questioned, as no causal relationship between CCSVI and MS has been confirmed [24-27,29-49]. In addition, the controversy and debate around CCSVI has been fueled by the postulated therapeutic effect of venous insufficiency correction using endovascular procedures [21], without first determining a real need for the procedure itself and testing its safety and efficacy in properly designed randomized, controlled and blinded trials [28,50,51].

Given that substantial resources by various governments and funding agencies have been devoted to studying the concept of CCSVI, it was recently proposed that funding of CCSVI research should be immediately abandoned because it is a waste of valuable time, money and intellectual energy [52-56]. Nevertheless, the concept of CCSVI has triggered an intense and rapid accumulation of knowledge over the past four years and has stimulated the need for further research to better understand the function and potential role of the extracranial venous system in CNS disorders and aging [57].

This review article highlights the need for better classification of extracranial venous abnormalities/developmental variants that is independent of any single diagnostic imaging modality. It also examines the anatomy, etiology and pathophysiology associated with venous abnormalities, as well as the clinical correlates in relation to various CNS disorders and aging.

## Anatomy of the extracranial venous system

In order to understand the potential role of the extracranial venous system in diseases of the CNS and aging, it is important to first appreciate the structure and function of the cerebral venous drainage system. Because this system is complex and poorly understood, in this section, a brief overview of the relevant anatomy is presented to assist the reader.

Cerebral circulation encompasses both the arterial and venous systems. The venous system contains approximately 70% of the blood volume, with approximately three-quarters of it within small veins and venules [58-64]. It is a system that is often asymmetric and considerably represents a more variable pattern than the arterial system [5].

### Cerebral venous system; superficial and deep veins

**Figure 1:**
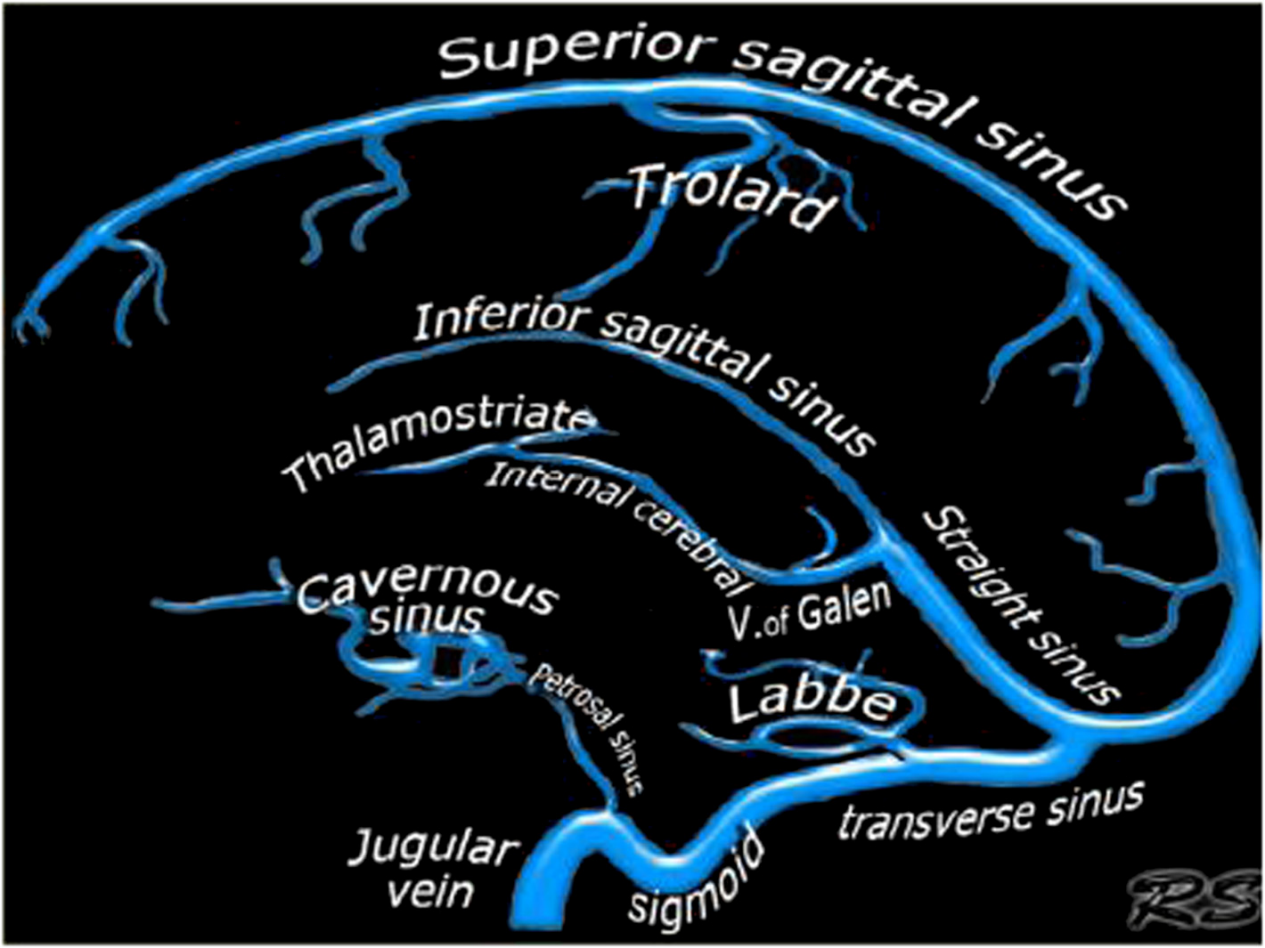
**Intracranial venous system anatomy of dural sinuses, cortical veins, deep intracerebral veins and cavernous sinus.** The figure was reproduced with permission from the Radiology Assistant website: (http://www.radiologyassistant.nl/en/p4befacb3e4691/cerebral-venous-thrombosis.html).

The venous drainage from cerebral hemispheres consists of two systems; the superficial and the deep venous system (Figure 1) [60-64]. The superficial system drains blood from the cortex and superficial white matter (WM) by cortical veins, collected by dural sinuses. There are two important dural sinuses: the superior sagittal sinus (SSS) draining dorso-laterally and the cavernous sinus draining anteroventrally. The transverse sinus then drains the SSS equally on both sides in only 20% of cases and asymmetrically in more than 50% of cases, depending on the configuration of torcular Herophili [60,63]. In 20% of cases, one transverse sinus drains the SSS in total (most often on the right side) and the other one drains the straight sinus, which collects blood from the deep venous system [63]. The cavernous sinus extends from the superior orbital fissure to the petrous apex, which receives orbital venous and middle cranial fossa drainage. From the cavernous sinus, blood drains posterolaterally along the superior petrosal sinus into the transverse sinus and inferior-laterally along the inferior petrosal sinus into the sigmoid sinus.

The deep cerebral venous system drains the deep WM and the regions surrounding the lateral and third ventricles or the basal cistern [60-62,65]. Three veins unite just behind the interventricular foramen of the Monro to form the internal cerebral vein(s). These include the choroid vein, septal vein and thalamostriate vein. The vein of Galen is a short (1 to 2 cm long), thick vein that passes posterosuperiorly behind the splenium of corpus callosum in the quadrigeminal cistern. The vein of Galen receives the internal cerebral vein, the basal veins of Rosenthal and the posterior fossa veins and then drains to the anterior end of the straight sinus where this unites with the inferior sagittal sinus. The main collecting vein for the deep venous system is the straight sinus, which receives the venous blood from the vein of Galen and flows into the transverse sinus (most often into the left side). The basal vein of Rosenthal is an important collateral pathway for the internal cerebral veins and the vein of Galen. By connecting with the superficial Sylvian vein via the deep Sylvian vein, venous blood flow can bypass the straight sinus.

Venous drainage of the posterior fossa mainly depends on the galenic system and the petrosal system and to a lesser extent, the tentorial veins and the transverse sinuses [60-63]. Therefore, factors influencing galenic system drainage would lead to venous congestion in both posterior fossa and brain regions drained by the deep venous system.

### Extracranial cerebral venous drainage pathway - neck veins

**Figure 2:**
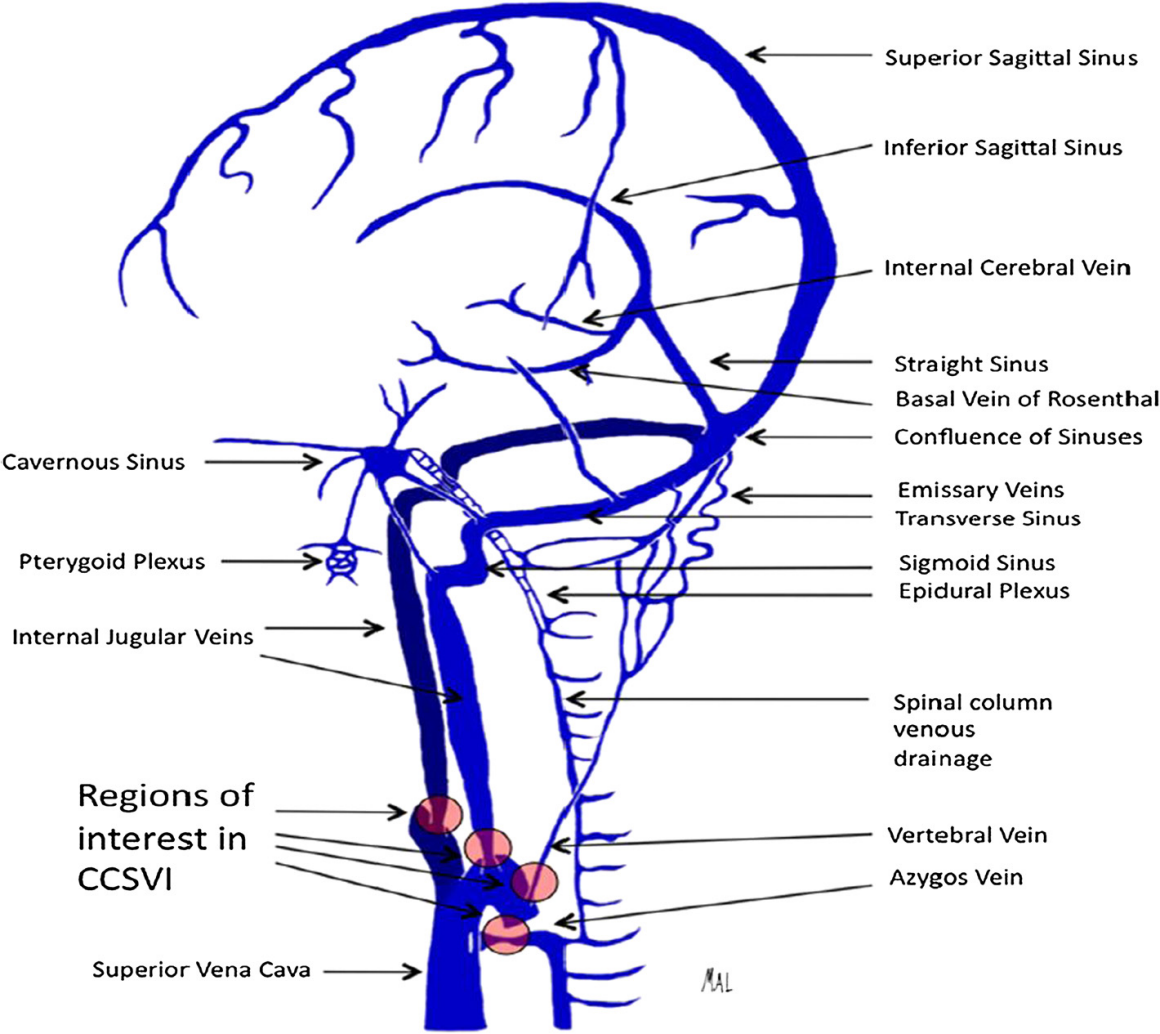
**Illustration depicting the predominant veins and sinuses involved in the craniocervical venous outflow.** Venous narrowing is depicted at locations of interest in chronic cerebrospinal venous insufficiency. The figure was reproduced with permission from Lazzaro MA, Zaidat OO, Mueller-Kronast N, Taqi MA, Woo D. **Endovascular therapy for chronic cerebrospinal venous insufficiency in multiple sclerosis.***Front Neurol 2011, ***2:**44.

Most of the cerebral venous drainage is via neck veins; mainly the internal jugular vein (IJV), vertebral venous system and deep cervical veins (veins in cervical soft tissue) (Figure 2) [66-70]. Consequently, there is a good reason to believe that impaired extracranial venous drainage functions or structures might cause cerebral venous drainage insufficiency and consequent neurological deficits.

The IJVs are the largest veins in the neck and are generally considered to be the most important cerebral venous outflow pathways. Venous drainage of the superficial and deep cerebral venous system is via the transverse sinuses to the sigmoid sinuses, which then drain into the IJV. The inferior petrosal sinus, a major drainage route collecting blood from the cavernous sinus, communicates with the basilar plexus, anterior and lateral condylar veins, anterior condylar confluence and vertebral venous plexus before draining into the IJVs [68,71,72]. The IJVs then join with the subclavian veins to form the brachiocephalic vein (BV). The confluence of the bilateral BV is the superior vena cava, which ultimately drains venous blood into the heart. Several tributaries in the neck also drain into the IJVs [73-75]. These bilateral IJV branches will interconnect with each other at the midline to form anastomosing plexi that can serve as collateral channels to maintain adequate venous drainage when the principal pathways are obstructed [73,74].

The vertebral venous system consists of two components; one is the vertebral venous plexus and the other is the vertebral vein (VV) [8,68,76,77]. The vertebral venous plexus can be subdivided as internal (posterior and anterior internal vertebral plexus) and external (posterior and anterior external vertebral plexus) [8,68,76,77].

Complex connections of cerebral venous outflow with the vertebral venous system over the craniocervical junction have been displayed by several human cadavers and angiographic studies [66,68,71,78-80]. The IJVs can also exhibit anastomosis with the other extracranial venous drainage system within the craniocervical junction region, which includes the anterior condylar confluent (ACC) and its tributes. Numerous anastomoses of the ACC make it a crossroad between the cavernous sinus, dural sinuses of the posterior fossa, IJVs and posterior cervical outflow tract (vertebral venous system and deep cervical veins).

#### IJV valves

The IJV valves make IJV a buffer zone between large central veins and the cerebral venous system. Although there are anatomical variations, the valves are generally located about 0.5 cm above the union of the subclavian vein and IJVs at the lower limit of the jugular bulb [81-85], which are shown in 96.8% of the general population [82,84]. The IJV valves are generally thought to prevent the backflow of venous blood and backward venous pressure into the cerebral venous system during conditions where the central venous pressure or intrathoracic pressure is increased, such as chest compression during external cardiopulmonary resuscitation, severe or repetitive cough and straining [81,83-86]. The pressure gradient across competent IJV valves can be as high as 100 mmHg [86]. Without competent IJV valves, a sustained or prolonged retrograde-transmitted venous pressure via IJVs might impair cerebral venous drainage and lead to neurological deficits. For example, IJV valve incompetence has been associated with encephalopathy after cardiopulmonary resuscitation [81,83-85].

#### Other neck veins serving as collaterals for cerebral venous drainage

Collateral veins probably represent physiological variations of the venous system that may play a compensatory role when there is narrowing of the principal pathways of the extracranial venous system [2,5]. The extra-jugular cerebral venous drainage system for cerebral venous drainage mainly consists of the vertebral venous system and deep cervical veins [22,36,66-70,87-91]. The external jugular vein (EJV) and anterior jugular vein (AJV), compared with the IJV, are located superficially in the neck. They serve as collaterals and become prominent (enlarged lumen) when the main cerebral venous drainage pathways (IJV and VV) are compromised [92,93]. EJV is formed by the confluence of the posterior branch of the posterior facial vein and the posterior auricular vein. It usually terminates into the confluence of the subclavian and IJV [94]. The AJV receives blood from superficial veins, such as EJVs, facial veins or IJVs. They usually end in the subclavian vein or EJV [94]. Bilateral AJVs may communicate via the jugular venous arch (JVA), which is located just above the sternum. The JVA receives tributaries from the thyroid gland via inferior thyroid veins [95,96]. In summary, venous collaterals in the neck include the anterior (jugular venous system) and the posterior (vertebral and other deep neck venous system) and different patterns of collateral establishment may reflect the location and severity of venous outflow obstruction.

### Extracranial cerebral venous drainage pathway - abdominal and thoracic veins

**Figure 3:**
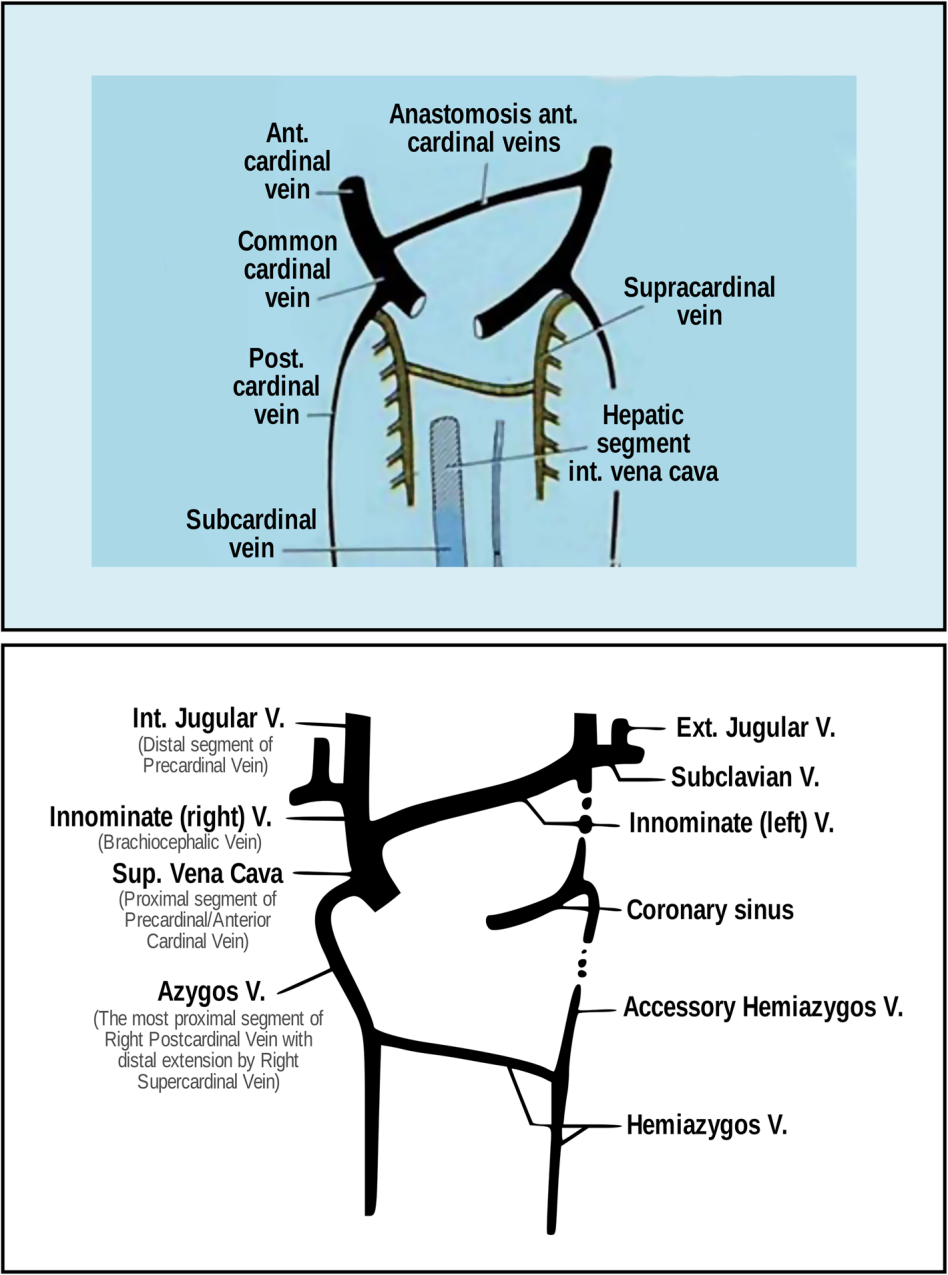
**Paired anterior cardinal veins form common cardinal veins with paired posterior cardinal veins, draining centrally into the sinus venosus (sinus horns) as depicted (top).** Paired anterior cardinals soon form an anastomosis between them; the connection grows from the left to the right anterior cardinal vein to form the left brachiocephalic (innominate) vein (bottom). The left anterior cardinal vein distal (cranial) to the anastomosis becomes the ‘left internal jugular vein,’ while the left anterior cardinal vein proximal to the brachiocephalic anastomosis regresses/atrophies to become the base of the ‘coronary sinus’ of the heart as displayed. The right anterior cardinal (precardinal) vein proximal to the right brachiocephalic vein forms the superior vena cava (SVC) with the common cardinal, and terminal/proximal segment of the posterior cardinal (postcardinal) vein. The figure was reproduced with permission from Lee BB: **Venous embryology: the key to understanding anomalous venous conditions.***Phlebolymphology* 2012, **4:**170–181.

The vertebral venous system, which is a rich plexus, communicates with the deep thoracic and lumbar veins, intercostal veins as well as the hemiazygos and azygos veins [10]. Abnormalities in these abdominal and thoracic veins may impair venous drainage from the vertebral venous system, which serves as an important collateral for cerebral venous drainage. The hemiazygos arch is connected with the left renal vein that represents a major outflow route for shunting blood into the inferior vena cava [10]. Ultimately, the azygos vein serves as the final venous blood collector and drains into the superior vena cava. The anatomy and developmental stages of the abdominal/thoracic blood vessels can be quite variable (Figure 3). For example, in some rare variations, the azygos vein also drains thoracic veins, bronchial veins and even gonadal veins. The vein is so named because it has no symmetrically equivalent vein on the left side of the body.

## Extracranial venous abnormalities and their etiology

A range of abnormalities have been reported in the extracranial veins that drain cerebral venous blood flow. These can be classified as structural/morphological, hemodynamic/functional and those determined only by the composite criteria and use of multimodal imaging. For example, structural/morphological venous abnormalities can be divided into those creating narrowing or occlusion and those causing abnormal distensibility. On the other hand, hemodynamic functional venous abnormalities represent an abnormal cerebral venous outflow in the presence or absence of a structural venous anomaly in the extracranial veins. Finally, because it is almost impossible to determine the relevance of a single structural/morphologic or hemodynamic/functional venous abnormality, regardless of the imaging modality or methodology utilized, the need for use of composite criteria by uni- or multi-modal imaging modalities of the extracranial venous system is emerging [2].

The etiology of these extracranial venous abnormalities is not well-defined. However, it has been hypothesized that these abnormalities may be related to embryologic developmental arrest, aging or other comorbidities [4].

### Structural/morphological venous abnormalities

The reason for the narrowing of extracranial veins can be intra-luminal or extra-luminal [10,11,37].

The venous system develops through stages that may be associated with a number of developmental variants that do not necessarily represent pathological findings [9-12]. Lee *et al.*[11] recently published a consensus document in which they proposed that most of the extracranial venous abnormalities are a result of congenital truncular venous malformations, which represent an embryologically defective vein where developmental arrest has occurred during the vascular trunk formation period in the ‘later stage’ of embryonic development [10]. For example, a truncular venous malformation lesion, such as a venous web at the hepatic venous outlet, causes portal hypertension, giving a profound damage/impact to the liver [10]. Similar truncular venous malformations involving the abdominal, thoracic and neck venous system may cause venous drainage impairment of the CNS. These truncular malformations are mostly represented by intra-luminal abnormalities.

Different extra-luminal etiologies lead to IJV narrowing/occlusion at different levels [2-4,22,26,37,48,90,91,97-102]. The sigmoid sinus exits the skull and becomes the upper jugular bulb, where it is directed anteriorly to sweep over the lateral arch of the first cervical vertebra. IJV narrowing at this level is commonly associated with displacement and compression of the vein as it courses over the anterior aspect of the lateral mass of the C1 vertebral body. At the mid-cervical level, IJV has been observed to be compressed by adjacent tissues, including carotid arteries and the sternocleidomastoid muscle group. The severity of these compressions could be dynamic, depending on the individual’s posture, neck flexion or extension and ipsilateral or contralateral rotation of the head position [3,103-105]. Most recently, the omohyoid muscle anatomic variants were reported as a possible reversible cause of IJV extrinsic compression [106].

It has also been proposed that the origin of these extracranial intra- and extra-luminal venous structural abnormalities could be acquired, due to inflammatory, viral, bacterial, cardiovascular, degenerative and aging processes [4,107-109]. In particular, it can be hypothesized that a lack of exercise, which is associated with higher disability present in numerous CNS diseases as well as in aging, may further contribute to the impairment of structural/morphological extracranial venous drainage pathways.

Pathological studies aimed to define the nature of these venous abnormalities or developmental variants are lacking [13,14]. Most recently, Diaconu *et al.* examined the IJVs, the BV and the azygos vein from 20 cadavers (10 controls and 10 MS patients) and concluded that the anatomy of the extracranial venous system has significant variability, including a differing number of valves in different regions and variable characteristics of the valves [14]. Coen *et al*. examined specimens from the IJVs of MS patients who underwent surgical reconstruction of the IJV, specimens of the great saphenous vein used for surgical reconstruction and specimens from patients without MS [13]. Focal thickenings of the wall associated with a higher expression of type III collagen in the adventitia was detected in specimens of MS patients. It could be hypothesized that this focal thickening of the venous wall is associated with the vein wall not reacting to a given change in transmural pressure. This phenomenon can be detected with various imaging modalities, as reduced distensibility/pulsatility/paradox.

#### Narrowing or occlusion of the venous drainage pathways

Restriction of the extracranial venous lumen may lead to abnormal narrowing, which represents a stenosis or even complete occlusion. The definition of “significant narrowing leading to stenosis of the major extracranial veins” is still arbitrary as no consensus guidelines are available at this time [2]. The lumen of the extracranial veins is not constant and may exhibit considerable variability, depending on anatomical location. Usually, the presence of significant narrowing or stenosis is defined as venous lumen reduction ≥50% respect to the proximal adjacent vein segment, on magnetic resonance venography (MRV), catheter venography (CV) and intravascular ultrasound (IVUS) [2,4,22,27,37,90,101,110-113]. However, the concept of a significant obstruction being when the vessel has been reduced to 50% of its diameter (which corresponds to a 75% reduction in cross-sectional area (CSA)) is derived mainly from observations in the arterial system [2]. Therefore, these criteria may not be applicable to the venous system as there are some fundamental differences between the two. In addition, the diameter of the veins varies with the anatomical level of the vein, particularly in the IJVs. Therefore, more sophisticated qualitative and quantitative criteria are needed to adequately assess the significant narrowing of the extracranial veins. Finally, further research is needed to determine whether the concept of significant narrowing corresponds to the hemodynamic consequences for the intra-cranial venous drainage, as recently reported [27,98,114]. For example, Traboloulsee *et al.*[27] recently proposed that a hemodynamically significant narrowing of the extracranial vein on CV is present, if at least one of the following criteria is recorded: 1) reflux (persistent retrograde flow of most of the contrast bolus after injection is completed); 2) stasis (contrast present 4 s after the injection); or 3) abnormal collaterals (one or more vessels >50% the size of the adjacent primary vessel or two or more collateral vessels present at <50% the size of the adjacent primary vessel).

Narrowing or occlusion of the extracranial veins can be observed at any level and the presence of multiple stenotic lesions is frequently observed [22,26,37,48,90,91,97-102]. By far, the most frequently identified site of IJV venous structural/morphological abnormalities is at the region of the jugular valve just cephalad to the internal jugular confluence with the BV [3,22,26,37,48,90,91,97-102]. In the azygos vein, the most common location of narrowing is at the level of the azygos arch [22,110].

Extracranial cerebral venous drainage pathway narrowing or occlusion is most frequently detected by single imaging modalities, including DS, MRV, CV or IVUS [2,4,97,113,115,116], although other non-invasive diagnostic techniques such as computed tomography venography and plethysmoghy are emerging as useful tools to study these abnormalities in a research setting [2,117-119].

##### Intra-luminal abnormalities

A intra-luminal structural/morphological abnormality is defined on DS as an echogenic structure extending from the endothelial lining of the vein wall with or without associated hemodynamic changes (reflux, decreased/no flow and so on) (Table 1) [5,22,37]. These include abnormal valves, web, multiple septa and/or flaps located in a cluster. Flaps can be defined as thin linear echogenic structures extending from the endothelial lining of a vein wall, while septum is a thin linear echogenic structure extending from the endothelial lining of a vein wall and attached to it at both ends. The septum may extend across a vein to attach on opposing sides or attach on the same side and the membrane shows as membranous structure almost occluding the entire diameter of the vein [37]. Webs represent multiple septae and/or flaps located in a cluster. In addition, Karmon *et al*. [110] described these on IVUS as intra-luminal hyperechoic filling defects and double parallel lumen of the veins. Various subtypes of malformed IJV valves have been reported, including fused leaflets, transverse leaflets, long leaflet, ectopic leaflet, accessory leaflet, inverted valves, sigmoid valves and double valves [14,27,37,102].

**Table 1:** Classification of the venous drainage pathways due to the extracranial structural/morphological, venous abnormalities

**Types **[10,11,22,27,37,110,111,115,120]	**Definition**
*Intra-luminal:*	• *Web:* multiple septae and/or flaps located in a cluster.
This is an echogenic structure detected by DS or by IVUS extending from the endothelial lining of the vein wall with/without the presence of functional abnormality. Use of a diluted angiographic contrast may help identification of these abnormalities on CV. These abnormalities include web, flap, septum, membrane, hyperechoic filling defect, double parallel lumen and malformed valve.	• *Flap:* thin linear echogenic structure extending from the endothelial lining of a vein wall.
	• *Septum:* thin linear echogenic structure extending from the endothelial lining of a vein wall and attached to it at both ends. The septum may extend across a vein to attach on opposing sides or attach on the same side.
	• *Membrane:* membranous structure almost occluding the entire diameter of the vein.
	• *Hyperechoic filling defect:* an eccentric hyperechoic crescent with a distinct sonographic signal, reminiscent of chronic organized thrombus.
	• *Double parallel lumen:* multiple small channels in the venous wall.
	• *Malformed valve:* dysdynamic or fibrous valve.
*Extra-luminal:*	• *Narrowing:* presence of significant narrowing (defined as venous lumen reduction ≥50% respect to the proximal adjacent vein segment on CV or CSA measurement of proximal IJV ≤0.3 cm^2^ on DS.
This is a restriction of the venous wall or narrowing detected on DS, CV, IVUS or MRV. These abnormalities include narrowing and annulus.	• *Annulus:* circumferential thickened vein wall that is restricting the vein from fully expanding with respiratory or positional changes.
	• Vein wall not reacting to a given change in transmural pressure on CV, IVUS or DS; non-compliant.
*Abnormal IJV distensibility/pulsatility/paradox:*	

Legend: CSA*,* cross sectional area; CV, catheter venography; DS, Doppler sonography; IJV*,* internal jugular vein; IVUS, intravascular ultrasound; MRV*,* magnetic resonance venography.

Intra-luminal venous abnormalities are found at proximal IJV just cephalad to the junction with BV by B mode of DS and IVUS [3,22,26,37,48,90,91,97],[99-102], while IVUS emerges as the most useful technique to detect intra-luminal abnormalities in the azygos vein [110-112,121]. There are no consensus guidelines with respect to the usefulness of CV for the detection of intra-luminal abnormalities. The recent position statement of The International Society for Neurovascular Disease (ISNVD) on the use of CV did not provide clear guidelines on this issue [115]. However, it has been reported that the use of diluted angiographic contrast may allow a better visualization of these intra-luminal structures (valve leaflets, webs and so on), while the non-diluted contrast allows a better opacification of epidural and other collaterals, as well as a better estimation of overall features of the veins.

In addition, it is very difficult to estimate the real contribution of intra-luminal abnormalities to significant narrowing, as they can be easily displaced by the catheter or by an inflated balloon and upon deflation, fall right back in to their original position and continue to obstruct flow. It is also unknown at this time what the variations of these abnormalities are with respiratory, positional and activity changes.

The role of intra-luminal abnormalities in venous drainage impairment has to be defined according to the temporal evidence of altered brain drainage due to these abnormalities. For example, Dolic *et al.* reported that the presence and number of intra-luminal IJV malformations were related to a higher number of collateral veins and functional abnormalities [37]. Of all intra-luminal abnormalities examined, the malformed valve (impaired mobility or thickened fibrotic valve), the septum and flap occurred most frequently in MS patients, as well as in healthy subjects [37].

The prevalence of intra-luminal abnormalities is not firmly established in the general population. Dolic *et al.* reported that a substantial number of MS patients (68%) and healthy subjects (49.2%) presented with at least one intra-luminal venous abnormality in their IJVs, as evidenced by DS [37]. In the Prospective Randomized Endovascular therapy in Multiple Sclerosis (PREMiSe) study, Karmon *et al.* found that intra-luminal abnormalities can be even more frequent in the azygos vein of MS patients (85%), as evidenced by IVUS. Further invasive studies are, therefore, required to investigate the prevalence of intra-luminal abnormalities in a variety of CNS diseases and the general population as well as their impact on the hemodynamic consequences of intra-cranial venous drainage.

##### Extra-luminal abnormalities

The extra-luminal structural/morphological abnormalities include narrowing and annulus (Table 1) [5,22,37]. As previously stated, the significant extra-luminal narrowing is considered a vessel that has been reduced to 50% of its diameter and that corresponds to a ≤0.3 cm^2^ of CSA proximal IJV measurement on DS in the supine position [22,37]. Annulus, a circumferential thickened vein wall that is restricting the vein from fully expanding with respiratory or positional changes, is another extra-luminal type of narrowing [22,37,102].

The prevalence of extra-luminal abnormalities has only been anecdotally investigated. Dolic *et al.* reported that 22% of MS patients and 11.1% of healthy subjects presented with narrowing ≤0.3 cm^2^ of CSA proximal IJV on DS in the supine position [37]. In another recent invasive study, Traboulsee *et al.* performed a CV in 79 MS patients and 98 healthy controls in which they investigated >50% narrowing of the IJVs (valvular or non-valvular vein segment) in comparison with a normal reference segment (widest vein segment below the mandible) in the supine position, and >50% narrowing of the azygos vein relative to the largest normal segment in the supine position [27]. Therefore, the >50% narrowing on the CV was not assessed respect to the proximal adjacent vein segment measurement. Using these criteria, they found that 74% of MS patients, 70% of healthy controls and 66% of unaffected siblings of MS patients had >50% narrowing on the CV in at least one of these three extracranial veins. In addition, they reported that 51%, 54% and 45% of these narrowing, respectively, created hemodynamically abnormal flow, as defined by the CV criteria [27]. Although this high rate of narrowing was described for the first time in healthy controls and while the authors concluded that venous narrowing is a common anatomical variant in healthy subjects, these data have to be interpreted with caution because of the narrowing criteria definition applied. Only longitudinal studies will be able to discern the real prevalence of extra-luminal abnormalities based on the demographic characteristics in different populations.

#### Abnormal IJV distensibility/pulsatility/paradox

Vessel compliance describes the extent to which volume changes in response to a given change in transmural pressure [122,123]. A venous wall not reacting to a given change in transmural pressure on CV, IVUS or DS is considered to be non-compliant (Table 1). Venous compliance was studied *in vitro* and *in vivo* by plethysmography [124], DS [26,37,125-130] and IVUS [110-112,116]. Those studies showed that large veins, compared with arteries, have a greater volume increment in response to increased transmural pressure, for example, a greater distensibility, within a wide-range of physiologic pressures.

Chung [120] used DS to measure the change in the vessel-lumen area of IJV during different grades of Valsalva maneuver (VM), which increases transmural pressure in IJV [131] in patients with migraine and in healthy individuals. The venodilatation of IJV in response to each level of VM pressure in patients with migraine was significantly less than that in healthy individuals. The reproducibility of this method appears acceptable [120]. Dolic *et al*. measured frequency and the number of paradox (vein wall not reacting to respiratory phase, non-compliant) using DS between healthy individuals and MS patients and found a relatively low prevalence (<1%) of these venous abnormalities in both groups [37].

Karmon *et al*. [110] used IVUS to examine reduced respiratory pulsatility or normal pulsatility (presence or absence of expansion movements of the vein wall according to respiratory frequency (10 to 20/minute during deep inspiration and during VM)) to confirm the pathologic versus the physiologic nature of the vein narrowing. They found reduced pulsatility in 35% of right IJVs, 55% of left IJVs and 35% of the azygos vein in MS patients.

### Hemodynamic/functional venous abnormalities

The hemodynamic/functional abnormalities include venous reflux/bidirectional flow, abnormal flow, no flow and abnormal posture control of IJV flow (Table 2).

**Table 2:** Classification for the extracranial hemodynamic/functional venous abnormalities

**Types**[22,24,25,27,29,40,47,48,64,90,91,98,101,110,112,114,116,118,119]	**Definition**
*Venous reflux/bidirectional flow:*	
Valsalva maneuver induced jugular venous reflux:	• Valsalva maneuver-like activities which increase intrathoracic pressure may lead to IJV incompetence, known as jugular vein reflux and measured on DS or IVUS.
*Spontaneous venous reflux:	• Present on DS examination in the IJV and vertebral veins and for more than 0.88 seconds with the head at 90° and 0°; delayed emptying time on CV.
*Venous reflux in the intracerebral veins:	• Reflux/bidirectional flow on DS in the deep cerebral veins is defined as reverse flow for a duration of 0.5 s in one of the intra-cranial veins.
*Abnormal venous flow distribution in extracranial veins:*	• Measurement of blood flow, blood volume and blood velocity by using DS, MR phase contrast imaging, CV or IVUS.
*No flow in extracranial veins:*	• No flow on DS or IVUS or contrast noted in the vein on CV and MRV, despite deep breaths.
*Abnormal posture control of IJV flow:*	• A negative ∆CSA on DS represents the loss of the normal postural control; altered estimation of changes in venous capacitance and venous resistance by posture change on plethysmography.

Legend: CSA*.* cross sectional area; CV*,* catheter venography; DS*,* Doppler sonography; IJV*,* internal jugular vein; IVUS*,* intravascular ultrasound; MRV*,* magnetic resonance venography.

*The controversy regarding the methodological validity of these quantitative definitions for spontaneous and intracerebral venous reflux included recent position statements from the ISNVD [97], the European Society of Neurosonology and Cerebral Hemodynamics (ESNCH) [132] and review studies [7] that expressed considerable concerns regarding the accuracy of the proposed criterion.

The etiologies of continuous JVR include central venous obstruction, such as mediastinal goiter, mediastinal masses, aortic aneurysm or venous thrombosis (SVC syndrome) [133-136] and one special anatomic factor occurring on the left side. Left BV has a more obtuse angle and a longer length before joining the superior vena cava than the right BV. Additionally, the left BV goes through the narrow space between the sternum and the thoracic outlet arteries before entering the superior vena cava. It may be that this narrow space can compress the left BV, causing narrowing of the lumen or even occlusion, resulting in left spontaneous JVR [137-139]. A higher frequency of JVR in the elderly may be due to the more-frequent engorged thoracic outlet arteries in this population [138].

VM-induced JVR, for example, IJV valve incompetence, is frequently seen in situations which have an elevated central venous pressure, such as congestive heart disease, tricuspid valve regurgitation, primary pulmonary hypertension and chronic obstructive pulmonary disease [86,140,141]. These conditions with chronic elevated venous pressure may damage the IJV valve gradually and make them incompetent. As with spontaneous JVR, VM-induced JVR is found more frequently at an older age [85,142].

#### Venous reflux/bidirectional flow

Venous reflux has been observed in the IJV, JV branches, VV, the azygos vein and in the intracerebral veins (basal veins of Rosenthal, superior and inferior petrosal sinus, and cavernous sinus, superior ophthalmic vein) by use of DS [19,20,24,26,33,40,64,97],[143,144].

##### Valsalva maneuver induced jugular venous reflux

Venous reflux in IJV (JVR) is the most commonly found venous hemodynamic abnormality which has been associated with certain CNS disorders. The pressure gradient determines the direction of flow in the veins [60]; therefore, JVR indicates an abnormal (reversed) pressure gradient resulting from increased venous pressure proximally [64]. When JVR results from elevated venous pressure proximal to the IJV valve, it is also known as IJV valve incompetence [86]. In physiological situations, the most frequently encountered reversed pressure gradient is due to VM-like activities which increase intrathoracic pressure. These activities include coughing, defecating, sexual intercourse and heavy lifting, and so on. During these activities, JVR will happen if the IJV valve is incompetent. This kind of JVR could be detected by DS and IVUS during VM [64,110]. Generally, VM-induced JVR is found more in the right IJV than in the left one [85,120]. In a large IJV hemodynamic registry with a wide age range from a healthy population, the mean prevalence of VM-induced JVR is 26% and 12% in the right and left IJV, respectively [120]. There is a higher frequency of VM-induced JVR in the elderly [85,142]. In people younger than 40 and older than 70 years old, the prevalence of VM-induced JVR is 18% and 30%, respectively, in the right IJV, and 6% and 26%, respectively, in the left IJV [120]. Patients with a chronic elevated central venous pressure, such as congestive heart disease [86,140], tricuspid valve regurgitation [86,139], primary pulmonary hypertension [140] and chronic obstructive pulmonary disease [141], also have higher frequency of VM-induced JVR.

##### Spontaneous venous reflux

Besides VM-induced JVR, there is another kind of JVR, spontaneous JVR, which is detected spontaneously at rest. Central venous obstruction and dural arterio-venous fistula (AVF) should be considered in individuals with a continuous JVR. The causes of central venous obstruction producing continuous JVR include goiter, mediastinal masses, aortic aneurysm and venous thrombosis (superior vena cava syndrome) [133-136]. Furthermore, continuous JVR is mostly reported on the left side because of the anatomic characteristics of the left BV that drains the left IJV [137,138,143]. This phenomenon is reported in normal individuals with a frequency of 0.2 to 0.4% [137,139]. Left JVR caused by this anatomic factor could reflux into the cerebral venous system as high as the level of basilar plexus via sigmoid sinus, transverse sinus and inferior petrosal sinus [143]. If there is another etiology for spontaneous, continuous JVR and for spontaneous intermittent JVR, it would need further evaluation.

Recently, Zamboni *et al*. introduced a quantitative definition of spontaneous venous reflux/bidirectional flow in the IJVs and/or in the VVs in sitting and in supine positions, as flow directed towards the brain for a duration of >0.88 s and incorporated it as one of the five venous hemodynamic (VH) criteria for the diagnosis of CCSVI. Using these criteria, Zamboni *et al.* investigated 65 MS patients and 235 controls composed, respectively, of healthy subjects, healthy subjects older than MS patients, patients affected by other neurological diseases and older controls not affected by neurological diseases but scheduled for CV by means of DS. They reported that 77% of MS patients and 0% of healthy controls (odds ratio 1,123) presented with spontaneous venous reflux/bidirectional flow in the IJVs [22]. Using the same DS criteria, Zivadinov *et al.* reported that out of 289 MS patients and 163 healthy controls, 45% of MS patients and 20.2% of healthy controls presented with spontaneous venous reflux/bidirectional flow in the IJVs [26]. However, Doeep *et al.,* using the same DS criteria in a study involving 56 MS patients and 20 healthy controls, found that nobody presented with spontaneous venous reflux/bidirectional flow in the IJVs. The controversy regarding the methodological validity of the quantitative definition of spontaneous venous reflux has resulted in position statements from the ISNVD [97], the European Society of Neurosonology and Cerebral Hemodynamics (ESNCH) [132] and review studies [7]; all of which expressed considerable concerns regarding the accuracy of the proposed criterion. Zamboni *et al.* argued that the value of >0.88 s allows operators to differentiate between a physiologic and pathologic reflux, adopting this threshold value from a study that examined IJV valve insufficiency during a VM [145]. Valdueza *et al.*[7] questioned the validity of this approach because the reference values gained during a VM do likely not apply to situations where the flow measurements take place in resting conditions. Nevertheless, this criterion has been widely-applied in recent studies aimed at determining the prevalence of CCSVI in patients with MS (Table 2) [24-27,30-36,40-45,100,146].

One of the important limits of DS for the detection of venous hemodynamic functional abnormalities is that the azygos vein cannot be directly imaged. While the specificity for detecting VV reflux on DS is high, the sensitivity is relatively low [36]. In our opinion, there are currently no available noninvasive imaging methods that can depict venous reflux in the azygos vein. Therefore, further development of imaging techniques is needed in relation to the accurate detection of venous reflux in the azygos vein [2].

In addition, using CV, Trabolusee *et al.* showed that >50% of MS patients and healthy controls showed hemodynamically abnormal flow in their IJVs and azygos vein, although they did not specify what was the exact prevalence of spontaneous reflux [27]. Based on this conflicting information from invasive and non-invasive studies, there is a need to further investigate the real prevalence of spontaneous reflux according to the demographic characteristics in different populations, using both invasive and non-invasive imaging methods.

##### Venous reflux in the intracerebral veins

Zamboni *et al.* defined reflux/bidirectional flow in the deep intracerebral veins as reverse flow for a duration of 0.5 s in one of the veins and reported a prevalence of 54% in MS patients and 0% in healthy controls (Table 2) [22]. Zivadinov *et al.* reported a prevalence of 46.8% in MS patients and 12.7% in healthy controls [26], while Doepp *et al.* showed that no healthy controls and only one of 56 MS patients presented with this DS criterion.

The assessment of this criterion is particularly controversial because the quantification and direction of the blood flow in veins connecting the cortical veins with deep veins may vary considerably as a consequence of the physiologic inter-individual variation of the cerebral venous anatomy and methodological issues related to the use of DS [7,25,36,97,132]. To avoid this issue, more sophisticated imaging techniques like fusion imaging technology [147] and quality Doppler profiles (QDP) were recently proposed; however, validation and applicability of those approaches remain unclear at this time.

#### Abnormal venous flow distribution in the extracranial veins

The measurement of blood flow, as well as velocity and blood volume, could be potentially more reliable in assessing the degree of venous outflow obstruction in the extracranial venous system.

IJV drains most of the cerebral venous blood flow during supine position [8,60,67,69]. A DS study showed that a total jugular flow volume of more than two-thirds of the global cerebral arterial inflow volume is present in 72% of healthy individuals and that less than one-third of the global cerebral arterial inflow volume is found in only 6% of healthy individuals [70]. Mancini *et al*. used contrast-enhanced DS to assess cerebral circulation times (CCT) in MS patients and healthy subjects which showed that MS patients had a significantly prolonged CCT and more frequent retrograde flow in IJVs [40]. Doepp *et al*. [25] reported that the decrease of total jugular blood volume flow on switching to the upright position was significantly less pronounced in MS patients, leading to significantly higher blood volume flow in the latter position. The meaning of these findings needs to be further explored but they were interpreted as an important sign of cerebral venous abnormality [148].

Another way to determine abnormal flow in the extracranial veins is to use phase-contrast MR angiography (PC-MRI) in order to measure blood flow and velocity [98,114,149]. Haacke *et al*. reported an abnormal flow distribution of IJV in patients with MS [98]. A total jugular flow volume of less than two-thirds of the global cerebral arterial inflow (arterial/venous flow mismatch) was found more frequently than in the healthy individuals. Furthermore, in these MS patients, the arterial/venous flow mismatch in the IJV stenotic group was significantly greater than the nonstenotic group. Therefore, this phenomenon of arterial/venous flow mismatch could be indicative of structural abnormalities in the main extracranial venous drainage pathway.

Karmon *et al*. used CV to estimate emptying time in MS patients [110]. They found prolonged emptying time in MS patients with stenotic IJVs.

#### No flow in the extracranial veins

The absence of flow in the IJV or/and VV in both the supine and sitting positions is mostly demonstrated by DS studies [26,97,99,100]. For example, Zamboni *et al.* reported that 63% of examined MS patients and 3% of healthy controls fulfilled this criterion on DS [22], while Zivadinov *et al.* by using the same methodology found that only 10.4% of MS patients and 7.4% showed abnormal flow in the IJVs. A similar prevalence was found by Doepp *et al.,* who reported 8.9% of abnormal flow in MS patients and 5% in healthy controls [25]. MRV, IVUS and CV also have played an increasingly important role in diagnosing a lack of flow in the IJVs, VVs and azygos vein [21,30,35-37,47,48,90,91,101],[102,110,113,114,150].

#### Abnormal posture control of IJV flow

Extracranial venous drainage is position-dependent [8,60,67,69]. Extra-jugular venous pathways are responsible for cerebral venous outflow in the upright position when an IJV is collapsed due to both increased external pressure and decreased IJV venous pressure when upright [60,151]. A negative ΔCSA represents the loss of the normal postural control, denoting a positive finding. Zamboni *et al*. proposed an assessment of reverted postural control of the main cerebral venous outflow pathway by measuring the difference in the CSA of the IJVs in the supine and upright positions and reported a prevalence of 51% in MS patients and 11% in healthy controls [22]. A number of other studies showed a substantially lower prevalence of this phenomenon in MS patients and healthy controls [22,24-26,31,43,44]. Other techniques, like plethysmography have been proposed as methods for the assessment of venous obstruction based on an estimation of changes in venous capacitance and venous resistance by posture change [118,119].

### Venous abnormalities determined by composite criteria and multimodal imaging modalities

The venous system is a complex, low-pressure, freely communicating network of vessels that is often asymmetric and represents significantly more variability than extracranial arterial anatomy. Because of this, it is almost impossible to determine the relevance of any single reported finding or imaging modality criteria, when considered in isolation, regardless of the imaging modality or methodology utilized. Therefore, the use of composite criteria using uni-modal and multi-modal imaging modalities are emerging as potentially useful tools to identify and evaluate possible pathologies of the extracranial venous system (Table 3) [2,121].

**Table 3:** Classification for the extracranial venous abnormalities determined by composite criteria or use of multimodal imaging with relative compensatory mechanisms

**Types **[2,27,36,37,48,91,98,110,152]	**Definition**
*Venous abnormalities determined by composite criteria and multimodal imaging modalities:*	
CCSVI:	• A cutoff for CCSVI diagnosis classification consists of two or more abnormal DS VH criteria.
VHISS:	• VHISS is based on the sum of extracranial venous abnormality VH criteria based parameters measured for each of the five CCSVI criteria examined and is ranging from 0 to 16.
Multimodal imaging application for detection of extracranial venous abnormalities	• Use of multimodal imaging criteria on DS, MRV, CV and IVUS to determine a significant narrowing of extracranial venous system with hemodynamic consequences for the intracranial venous drainage.
*Compensatory mechanisms for venous abnormalities:*	
Collateral veins:	• The presence of two or more extracranial collateral veins and of epidural collateral veins may serve as an indirect sign of impaired venous outflow.

Legend: CCSVI, chronic cerebrospinal venous insufficiency; CV, catheter venography; DS, Doppler sonography; IVUS, intravascular ultrasound; MRV, magnetic resonance venography; VH, venous hemodynamic criteria; VHISS, venous hemodynamic insufficiency score.

#### Chronic cerebrospinal venous insufficiency

In 2009, Zamboni *et al*. coined the term CCSVI introducing four extracranial and one intracranial VH criteria [21-23]. The VH DS criteria include: (1) reflux present in an outflow pathway (IJV and/or VV) with the head at 0° and 90°; (2) reflux in the intracranial veins/deep cerebral veins; (3) high resolution B-mode evidence of proximal IJV narrowing and/or other B-mode anomalies; (4) flow not detectable in the IJVs and/or VVs despite numerous deep inspirations; and (5) abnormal posture control of IJV flow. CCSVI was described as a vascular condition characterized by anomalies of the main extracranial veins, mainly in IJVs and azygos veins that interfere with normal venous outflow from the brain to the periphery, being specifically associated with MS [21-23].

CCSVI implies a pathological condition or disorder which is diagnosed using color DS of the extracranial (neck) - and intracerebral (deep cerebral) veins. A cutoff for CCSVI diagnosis classification consists of two or more abnormal DS VH criteria [22,23]. The construct of the CCSVI cut-off is based on an arbitrary decision biased toward characteristics of the originally studied population and on the obtained results without further testing and validation of the datasets [22,23]. The categorical variable construct of the CCSVI diagnosis may contribute to explaining major inconsistencies in the prevalence of findings of CCSVI between different studies [22-26,29-34,40-42,45,49,100,146,153]. Zamboni *et al.* originally reported that of 109 MS patients studied, 100% presented with DS diagnosis of CCSVI, while of 177 healthy controls, 0% met the CCSVI DS criteria [23]. Zivadinov *et al.* used the same DS criteria and showed that 56.1% of MS patients and 22.7% of healthy controls met DS criteria for a diagnosis of CCSVI [26], while Doepp *et al.* found no MS patients and healthy controls fulfilled these criteria [25]. Most recently, Comi *et al.* performed a multicenter CoSMo study that involved 35 centers in Italy and evaluated 1,767 subjects, including 1,165 MS patients, 226 patients with other neurologic diseases and 376 healthy controls [153]. The prevalence of central CCSVI reading by three DS experts was 3.26% in MS patients, 3.1% in other neurological diseases and 2.13% in healthy controls. The overall CCSVI prevalence in the local readings was significantly higher, as compared to the first centralized reading (14.9% versus 3.2%; *P*<0.001) but there was no difference in the prevalence among the three study groups. Therefore, it can be concluded from these and other DS CCSVI studies [2] that given that multiple VH criteria are acquired, the reproducibility of the categorical CCSVI diagnosis depends on the training level, skills of the operator and reading criteria. Also to note, it is not easy to be blinded and standardized in either a research or clinical setting [36,153,154]. Because of this, usefulness and applicability of these criteria in clinical research and practice is limited.

While the CCSVI diagnosis construct is based only on the DS criteria, Zamboni *et al.* performed CV in their original study and confirmed their DS findings in 65 MS patients and 48 healthy controls [22]. They created the four patterns of venous obstruction, highly indicative of CCSVI, including narrowing of the proximal azygos vein and complete occlusion of one IJV (type A), narrowing of both IJVs and the proximal azygos vein (type B), bilateral narrowing IJVs only (type C) and azygos vein narrowing (type D). By using these CV patterns indicative of CCSVI, they were able to classify all MS patients into the particular CV patterns and none of the healthy controls [22]. Most recently, Traboulsee *et al.* performed a study that investigated the same CV patterns in 79 MS patients and 98 healthy controls and found that only 2% of MS patients, 2% of unaffected siblings and 3% of unrelated healthy controls presented with these CV CCSVI patterns [27].

Based on this and other evidence [2], the DS composite criteria-based diagnosis of CCSVI should be used with caution and cannot imply a pathological condition that requires an endovascular intervention. Screening and monitoring of the extracranial venous abnormalities using a combined non-invasive and invasive imaging approach should help establish the actual incidences and prevalence of this condition in various populations.

#### Venous hemodynamic insufficiency severity score

To create a more comprehensive quantitative measure indicative of the severity of extracranial venous system drainage impairment that is not biased by categorical construct, Zamboni *et al*. introduced the venous hemodynamic insufficiency severity score (VHISS). VHISS is based on the sum of extracranial structural and hemodynamic venous abnormality VH criteria based parameters measured for each of the five CCSVI DS criteria examined [152]. VHISS ranges from 0 to 16. In a number of recent studies, VHISS showed a better relationship with other clinical and MRI outcomes, than did the diagnosis of CCSVI [152,155-159]. For example, Weinstock-Guttman *et al.* showed that a CCSVI DS diagnosis was not associated with disability, as measured by the Expanded Disability Status Scale (EDSS) in MS patients, while the VHISS was related to the EDSS subscores [155]. Therefore, quantitative composite criteria which reflect the total amount of extracranial venous abnormalities may be more useful in predicting clinical and other imaging outcomes in CNS disorders and aging than the categorical ones.

#### Multimodal imaging application for detection of venous abnormalities

The discrepancy in the prevalence of extracranial venous abnormalities between different studies using non-invasive and invasive imaging techniques [22-26,29-34,40-42,45,49,100,146] emphasizes the urgent need for the use of a multimodal imaging approach for better understanding of these venous abnormalities and developmental variants [2]. The prevalence of venous abnormalities of the extracranial venous system is even higher, when investigated with sophisticated invasive imaging techniques [27,110-112,116]. A multi-modal imaging approach is recommended to determine the range of venous abnormalities and anatomic variants and to what extent they are present in various healthy and disease groups as well as disease conditions [2]. Creation of multimodal imaging quantitative criteria that will incorporate structural and hemodynamic findings to describe extracranial abnormalities is the most important step toward understanding what is physiological and what is pathological.

### Compensatory mechanisms for venous abnormalities

From a biomechanical point of view, the presence of collateral flows is the strongest evidence for constricted principal venous pathways and venous hypertension. This is because increased up-stream blood pressure is required to open up (inflate) the collateral veins, by overcoming the elastic forces in the endothelia which would normally mean that the lumen of these vessels remains narrow. In subjects with IJV narrowing, prominent extra-jugular veins serving as collaterals have been demonstrated in many studies [22,27,37,87-91,121]. While healthy individuals regularly present with extracranial venous collateral circulation, the presence of two or more collateral neck veins most likely represents a compensatory mechanism for impaired venous outflow because it bypasses blocked veins and thereby reduces resistance to drainage [27,36,37]. The use of CV and MRV represents an excellent way for the assessment of the possible prominence or collateralization of the extracranial neck veins [2].

Thoracic epidural collateralization was observed in MS patients with a narrowing (detected by IVUS or CV) [22,110,121]. The existence of collaterals in cases with no observed azygos vein narrowing may stem from the presence of intra-luminal abnormalities that are evident on IVUS but not on CV [110,121]. The presence of venous abnormalities may disrupt anterograde flow long enough that collaterals are recruited to compensate. Moreover, the presence of these extensive epidural collaterals may reflect venous hypertension in the cervical and thoracic spinal cord, a hallmark of the CCSVI hypothesis [110,121].

## Pathophysiology of extracranial venous abnormalities (theories and current evidence)

Studies and observations of diseases with inadequate cerebral arterial supply are extensive compared with those related to cerebral venous drainage disorders. The poor understanding of the pathophysiology may consequently underestimate the impact of cerebral venous drainage abnormalities in a variety of CNS disorders [7,60,156]. Consequently, there is a need for more basic science and clinical studies to increase our knowledge and understanding of the clinical association and pathophysiologies of cerebral venous drainage abnormalities. Here below, we report some of the presumed theories and current available evidence regarding the pathophysiologies of extracranial venous abnormalities.

### Decreased cerebral perfusion by increased cerebral venous pressure

An obstruction of the extracranial venous drainage pathways may reduce the supply of brain nutrients and potentially result in hypoxia. A hypoxia-like condition has been evidenced in patients with many neurodegenerative diseases, including MS. Therefore, local blood congestion and secondary hyperemia of the brain parenchyma may be related to extracranial venous hemodynamic abnormalities that result in increased cerebral venous pressure [157]. Nevertheless, it is not clear at this time whether reduced perfusion of the brain parenchyma in MS patients is a sign of vascular pathology, decreased metabolic demand [158] or precipitated hemodynamic changes in the extracranial venous pathways [159,160].

#### Jugular venous reflux

Retrograde flow detected in IJV, for example, JVR, might cause cerebral venous drainage impairment. Without a competent IJV valve or with venous pressure higher than IJV valve’s competence, JVR will occur [64,157]. The elevated venous pressure would cause retrograde transmission through IJVs into the cerebral venous system, which may increase cerebral venous pressure and then decrease cerebral perfusion pressure and cerebral blood flow (CBF), leading to cerebral venous ischemia [38,64,86,157,161,162]. The exact magnitude of increased cerebral venous pressure that would lead to altered CBF is unknown at this time. For example, Meyer-Schwickerath *et al.* investigated intracranial venous pressure by using ophthalmodynamometry in 29 MS patients, 28 healthy subjects and 19 cases with elevated intracranial pressure and found no evidence of increased intracranial pressure in MS patients or healthy controls [163]. On the other hand, Beggs *et al.* reported that rapid discharging of the contents of the cortical veins might lead to a transient increase in pressure in the SSS of patients with MS [118]. More research is needed to elucidate whether extracranial venous abnormalities may lead to increased venous pressure in the SSS.

After several clinical observations concerning JVR, Chung and Hu [17,18,20,64,120,142-144,162,164],[165] have made efforts to provide more evidence supporting the theory that retrograde transmission of venous pressure by JVR has an impact on cerebral circulation. They studied healthy individuals and found that subjects with VM-induced JVR have wider retinal venular diameters and higher CBF decrement during VM compared to subjects without JVR [164,165]. These results imply that retrograde transmission of venous pressure by JVR could reach the cerebral venous system and decrease CBF respectively. They have also established an animal model of JVR to elucidate a more detailed pathophysiology of JVR [166].

There is other evidence supporting the theory that JVR can cause harm to cerebral structures, especially to the WM [18,167-169]. Clinical reports of unilateral dural AVF with venous reflux from sigmoid sinus could produce bilateral diffuse cerebral WM abnormalities on MRI and hypoperfusion in these WM abnormalities on single-photon emission computed tomography [167-169]. Another clinical study of aged people also showed that the severity of age-related WM abnormalities (leukoraiosis) is associated with the severity of JVR which is not caused by AVF [18].

Even in dural AVF, an additional precipitating factor, such as contralateral venous outflow obstruction, would be needed to exacerbate the severity of cerebral venous congestion and neurological deficits [170-172]. For example, JVR needs other precipitating factors, which would cause cerebral vascular abnormalities, to be able to correlate with the severity of age-related WM abnormalities [18]. The association between the presence of JVR and cough syncope is strengthened when there is an elevated level of circulatory endothelin 1, on which a strong vasoconstrictor may synergistically act on cerebral vessels and perfusion [16].

#### Extracranial venous drainage obstruction

There are only a few clinical studies to evaluate the impact of extracranial venous drainage obstruction on cerebral circulation. Bilateral occlusion of IJV in infants has shown a decrease of extracranial artery inflow, most likely due to increased cerebral venous pressure and decreased perfusion pressure [171]. Rat models with bilateral jugular vein occlusion showed a reversible decrease of CBF and no histopathological changes in the brain; however, this study only observed the effects within one week [172]. A recent study used SJL mice with bilateral jugular vein ligation and the mice were observed for up to six months after ligation [170]. Sham-operated mice and mice induced with experimental autoimmune encephalomyelitis were used as negative and positive controls, respectively. The authors did not identify changes in the brain–blood barrier (BBB) permeability, neuroinflammation, demyelination or clinical signs in the jugular vein ligation group compared to the sham group. Whether or not it does and how cerebral extracranial venous drainage pathway obstructions, such as narrowing/occlusion, influent cerebral circulation and structures contribute to the problem need more study.

Since prominent venous collaterals appear after occlusion of the principal venous drainage pathways in human and animal studies [22,27,37,69,76,77,87-91,98], it is reasonable to postulate that the capacity for the establishment of collaterals might play an important role in determining the impacts of extracranial venous drainage obstruction on cerebral circulation and structures.

As in JVR, additional precipitating factors may be needed in addition to extracranial venous drainage obstruction, in order for pathological effects to occur. For example, IJV compression by the lateral arch of C1 vertebra would cause cerebellar venous congestion and hemorrhage only under a long-term posture (head rotation to contralateral side with neck extension) for unilateral supratentorial craniotomy [103].

### Cerebral microvascular damage by cerebral venous hypertension

Cerebral venous hypertension would cause microvascular abnormalities, such as impaired arteriolar autoregulation and endothelial function, BBB damage, venular wall thickening, hyalinosis and possibly iron deposition [169,173-179]. To demonstrate whether extracranial venous drainage obstruction may elevate cerebral venous hypertension and lead to these microvascular abnormalities would need further studies. However, Beggs [157] and Dake *et al*. [3] postulated that extracranial venous drainage abnormalities may increase cerebral venous pressure and consequently cause microvascular endothelial activation as well as BBB damage, which might favor autoimmune leukocyte accumulation in cerebral vasculatures and invasion into the brain. This presumption would support that extracranial venous abnormalities may play a potential role in the pathophysiology of CNS disorders.

### Altered cerebrospinal fluid flow dynamics, as consequence of impaired extracranial venous drainage

Normal cerebrospinal fluid (CSF) circulation, in which homeostasis is maintained between the ultra-filtration of CSF (in the veins of the lateral ventricles) and clearance into the venous system at the level of the dural sinuses, depends on efficient extracranial venous drainage. Any occlusion of the extracranial venous drainage pathways is likely to induce hypertension in the venous sinuses [40]. Increased pressure in the SSS can inhibit the absorption of CSF through the arachnoid villi, decrease CSF brain parenchyma drainage and induce hypoxic stress in the endothelia [180]. Moreover, after reopening of the extracranial veins drainage pathways by means of venous angioplasty in MS patients, significant improvement in the CSF flow were detected [181].

A recent hydrodynamic analysis by Beggs summarizes the relationship between extracranial venous abnormalities and increased CSF pulsatility dynamics and decreased CBF changes intracranially, which are commonly observed in conditions like leukoraiosis, normal-pressure hydrocephalus (NPH) and MS [157]. Given that NPH is associated with venous hypertension in the dural sinuses [182], it may be that impaired cerebral venous outflow alters the dynamics of the intracranial CSF system, irrespective of any pathology. In order to evaluate whether or not CCSVI is associated with changes in the dynamics of the intracranial CSF system, Beggs *et al.* undertook a study involving 51 age-matched healthy individuals (32 CCSVI negative and 19 CCSVI positive subjects) with no family history of MS [183]. They found that net positive CSF flow was 32% greater in the CCSVI positive group compared with the CCSVI negative group indicating that CSF dynamics are altered in CCSVI positive healthy individuals, as demonstrated by increased pulsatility. This finding was accompanied by enlargement of the CSF spaces, suggesting that structural changes may be occurring in the brain parenchyma of CCSVI positive healthy individuals.

A recent article reported that natural sleep or anesthesia is associated with an increased flushing of the toxic material from the CNS, suggesting a new biological purpose for sleep [184]. The authors found a 60% increase in the interstitial space during sleep, resulting in a striking increase in convective exchange of CSF with interstitial fluid. Alzheimer’s disease (AD), the most common form of dementia in the elderly, is thought to be caused by an imbalance between amyloid-β (Aβ) production and clearance leading to Aβ accumulation in the CNS, which then causes neuronal damage and death manifesting as progressive clinical dementia [185]. Patients with AD have a 30% slower clearance of Aβ [18]. One of the possible etiologies of decreased Aβ clearance may be related to decreased CSF flow due to narrowing of the extracranial venous system pathways, as recently suggested [183,186].

Because the venous drainage of the CNS is mostly driven by the IJVs in the supine position, the relationship between CSF flow clearance and the presence of extracranial venous abnormalities should be further explored in aging and neurodegenerative disorders.

### The role of precipitating risk factors for the extracranial venous abnormalities

Exploring the role of precipitating risk factors for extracranial venous abnormalities may help elucidate their pathophysiology [4,108,109]. Dolic *et al*. studied 240 healthy individuals and found that the presence of heart disease, especially heart murmurs, obesity and cigarette smoking were associated with an increased prevalence of extracranial venous abnormalities. In another study, including 252 healthy individuals, they reported that a history of infectious mononucleosis and irritable bowel syndrome was associated with a diagnosis of CCSVI [108]. While, these results may imply that acquired cardiac valvular disease-related hemodynamic changes and inflammation (autoimmune or infection) may be involved in the pathophysiology of venous structural and hemodynamic venous abnormalities; no causality can be established without conducting prospective longitudinal observational studies [4].

Evidence is mounting that the prevalence of extracranial venous abnormalities increases with aging [2,4]. However, at this time, it is not clear whether an incidence of these abnormalities may differ over the lifetime or in relation to the disease states. For example, Dolic *et al*. used DS and MRV to study extracranial venous abnormalities in the IJVs of 150 MS patients and 63 healthy individuals. They reported that different structural and hemodynamic venous abnormalities were observed at different stages of MS disease [37]. Based on these findings, they proposed a chronological development of venous abnormalities in which intra-luminal structure abnormalities develop first, followed by hemodynamic functional abnormalities and the development of venous compensatory response mechanisms (collaterals establishment). When this compensatory ability is overcome, extra-luminal abnormalities begin to form [37]. This theory is supported by a number of recent studies which found that extra-luminal venous abnormalities are very rare at MS disease onset but become more frequent in subjects with a longer MS duration [24,26,31,35,36,47]. However, longitudinal observational studies will need to be conducted in order to prove or disprove the dynamic of extracranial venous system changes over time.

### Decreased IJV distensibility in migraine

Large veins have a great distensibility in response to increased transmural pressure, which helps keep venous pressure within a normal physiologic pressure [125-130]. When IJV loses this compensation ability, it becomes prone to IJV venous hypertension, which might impair cerebral venous drainage or retrogradely transmit venous hypertension into cerebral circulation.

A decreased IJV distensibility in subjects with migraine was found compared to healthy individuals [120]. Trigger factors, such as stress, sleep deprivation and menstrual cycle, are frequently found in patients with migraine [187-189]. Certain triggering factors of migraine would increase the sympathetic tone which could increase the venous tone and pressure [190-192]. It has been postulated that less compliant IJVs in subjects with migraine have less ability to compensate and alleviate increased IJV pressure by these triggering factors and, therefore, increased IJV pressure might transmit into cerebral venous structures and lead to a headache attack [120].

## Associated central nervous system disorders and aging

A link between the presence and severity of extracranial venous abnormalities and several CNS disorders as well as aging are emerging. The described associations are mainly reported with JVR, CCSVI and abnormal distensibility vein conditions.

The central issue to be determined is whether structural/functional abnormalities and their developmental variations may play a potential role, as precipitating factors, in increased susceptibility for a number of CNS diseases.

### Associations with jugular venous reflux

Studies finding clinical associations between JVR and neurological disorders are emerging [64].

#### Inducible central nervous system disorders

CNS disorders induced by VM-like activities (for example, cough, straining and certain physical exercises, and so on) are found to be associated with VM-induced JVR (for example, IJV valve incompetence). These CNS disorders include transient global amnesia [17,143,193-196], transient monocular blindness [20], cough, headache [15], exertional headache [19] and cough syncope [16,197]. JVR during VM-like activities causes retrograde transmission of pressure into cerebral venous circulation and causes transient cerebral venous hypertension and decreased CBF in certain brain regions and relevant neurological deficits.

#### Age-related central nervous system disorders

Compared with inducible JVR, sustained JVR may cause sustained, elevated cerebral venous pressure and CBF decrement. Besides chronic hypoperfusion, chronic venous hypertension would cause venular wall thickening and activate inflammation in venular walls and perivenular tissues [178,198]. In image and autopsy studies of chronic cerebral venous hypertension, diffuse WM changes, BBB damage and perivenular demyelinating were noted [165-169,199-201].

Recently, it has been found that the severity of age-related WM changes (leukoraiosis) is related to the severity of JVR, especially lesions in caudal brain regions (the occipital, basal ganglia and infratentorial regions) [18]. As mentioned above, the frequencies of both spontaneous and VM-induced JVR does increase with age [85,138,142]. JVR with a sustained (in spontaneous JVR) or long-term repetitive (in VM-induced JVR) retrograde-transmitted venous pressure into cerebral venous system would cause harm to cerebral vasculatures and tissues, which may accumulate with aging and lead to age-related chronic cerebral hypoperfusion and consequently WM abnormalities [162,164,165]. Most recently, Chung *et al.* investigated whether JVR is associated with cerebral WM changes in 12 individuals with AD, 24 with mild cognitive impairment (MCI) and in 17 elderly age- and sex-matched controls [186]. The results of this study suggested that there may be an association between JVR and WM in AD patients, implying that cerebral venous outflow impairment may play a role in the dynamics of WM changes/formation in AD patients, particularly in the periventricular regions. Whether or not JVR plays a role in other neurological diseases associated with age-related cerebral circulatory insufficiency, is a question to be answered in future longitudinal studies.

### Associations with chronic cerebrospinal venous insufficiency (CCSVI)

CCSVI was initially described in the context of MS [22,23]. It gained quick popularity among MS patients because of the postulated possibility of venous insufficiency correction using endovascular procedures [119]. However, it became clear with the first controlled studies that CCSVI is not the cause of MS and can be present in healthy individuals and patients with other neurologic diseases [24,25,33,41,42,159]. The major amount of knowledge regarding MS points toward immune etiopathogenesis [202]. A number of recent studies examined a cause-and-effect relationship between MS and CCSVI by applying the so-called Bradford Hill criteria [4,52,203]. The Bradford Hill criteria examines the strength of the association, the consistency, the specificity, the temporality, the biological gradient and plausibility, the coherence, the experiment and the analogy between the two phenomena [204]. In the case of MS and CCSVI, all of these criteria are partially or not fulfilled [4,52,203]. However, the precipitating role of extracranial venous abnormalities in facilitating immune attack, mediated by host-viruses in genetically predetermined individuals, cannot be excluded, in our opinion at this time completely, and should be further investigated. However, it could also be that reduced perfusion in MS patients may exert a precipitating role in inducing structural/functional changes of the extracranial venous system.

The CCSVI hypothesis has provoked great controversy and debate in the MS research community since it was first presented [28,50,52-57]. Whether CCSVI is a syndrome or condition that is primarily characterized by symptoms, such as headache, fatigue, sleep disturbances, autonomic dysfunctions and so on, that can be improved using endovascular treatment and possibly independent from the other underlying disease process is unclear at this time [51].

Many MS patients have undergone endovascular treatment for CCSVI procedures in either an open-label or private care setting [51]. The most important driver of this momentum has been a tremendous patient advocacy-based response in support of the widespread availability of venous angioplasty. Many patients with a desire to achieve a cure for this chronic, severely disabling malady have traveled far and wide to receive treatments from surgeons specializing in the venous angioplasty procedure. As with many yet unproven therapies, safety and efficacy concerns have been raised [54-56,205-214] without properly designed clinical trials [51,213,214]. An unknown number of MS patients have reportedly suffered serious adverse events, including stroke and death. For example, Ghezzi *et al.* reported in a retrospective study severe adverse events after endovascular treatment in 15 of 462 subjects (3.3%) at a variable interval after the procedure [211]. These included jugular thrombosis in seven patients, and tetraventricular hydrocephalus, stroke, paroxysmal atrial fibrillation, status epilepticus, aspiration pneumonia, hypertension with tachycardia or bleeding of bedsores in the remaining seven cases. One patient died because of myocardial infarction 10 weeks after the procedure. Therefore, the risk of severe adverse events related to endovascular treatment for CCSVI must be carefully considered.

A number of uncontrolled endovascular studies reported subjective physical and quality of life improvements in MS patients after endovascular treatment for CCSVI [213-216]. However, no objective evidence of improvement is available at this time [56]. It is our view that the association between CCSVI and MS can only be studied in blinded, randomized, controlled clinical trials that will assess the benefits of endovascular interventions according to established clinical (annualized relapse rate, sustained disability progression), MRI (lesion activity and brain atrophy) and quality-of-life treatment outcomes. However, only safe and ethical approaches should be encouraged in designing new clinical trials.

### Associations with abnormal extracranial vein distensibility

Chung and Hu [120] found that patients with common migraine have decreased venodilatation of IJV in response to each level of VM pressure compared with healthy individuals, which may play a role in the pathophysiology of migraine [120].

## Conclusions and perspectives

The classification of the presence and severity of extracranial venous abnormalities/developmental variants by imaging and pathology findings should be the first step in the determination of their role in the pathology of CNS disorders and aging. The extracranial venous abnormalities could be classified as structural/morphological, hemodynamic/functional and those determined only by the composite criteria and use of multimodal imaging.

One of the central issues to be further investigated is the definition of significant narrowing leading to stenosis of the major extracranial veins. The current definition (narrowing of >50%) respect to the proximal adjacent vein segment is mainly derived from observations in the arterial system. Even more important is to establish what constitutes a significant narrowing of extracranial venous system with hemodynamic consequences for the intracranial venous drainage. More sophisticated and validated quantitative single or composite multimodal imaging criteria are needed to adequately assess the clinical impact of significant narrowing with hemodynamic consequences of the extracranial veins for the CNS pathology. Because disruption of normal flow is associated with prominent collateral circulation as the main compensatory mechanism, this has to be taken into account when determining the impact of significant narrowing.

The etiology and pathophysiology of extracranial venous abnormalities in relation to aging or the development of other CNS comorbidities should be further investigated. Pathological and imaging approaches need to investigate the origin of extracranial venous abnormalities. It is necessary to determine the incidence and prevalence of extracranial venous abnormalities in relation to embryologic/developmental arrest factors, demographic factors (such as age, sex, race), cardiovascular risk factors (smoking, obesity, hypertension, diabetes, hyperlipidemia), inflammatory comorbidities and other possible precipitating risk factors, such as one’s level of exercise and diet. Only properly designed, safe and ethical studies should be encouraged in collecting this longitudinal observational information.

While some CNS disorders have been linked to the presence and severity of JVR and CCSVI, the ultimate cause-consequence relationship has not been firmly established. CCSVI triggered great interest and debate, highlighting the need for a better understanding of the role of extracranial venous abnormalities but many questions remain unanswered at this time. The use of endovascular treatment for the correction of these extracranial venous abnormalities should be discouraged until the potential benefit is demonstrated in properly-designed blinded, randomized and controlled clinical trials.

## Abbreviations

ACC: Anterior condylar confluent; AD: Alzheimer’s disease; AJV: Anterior jugular vein; AVF: Arterio-venous fistula; BBB: Brain–blood barrier; BV: Brachiocephalic vein; CCSVI: Chronic cerebrospinal venous insufficiency; CNS: Central nervous system; CSA: Cross-sectional area; CSF: Cerebrospinal fluid; CV: Catheter venography; DS: Doppler sonography; EDSS: Expanded disability status scale; EJV: External jugular vein; ESNCH: European Society of Neurosonology and Cerebral Hemodynamics; IJV: Internal jugular vein; ISNVD: International Society for Neurovascular Diseases; IVUS: Intravascular ultrasound; JVA: Jugular venous arch; JVR: Jugular venous reflux; MRV: Magnetic resonance venography; MS: Multiple sclerosis; NPH: Normal-pressure hydrocephalus; PC-MRI: Phase-contrast MR angiography; QDP: Quality Doppler profiles; SSS: Superior sagittal sinus; VH: Venous hemodynamic; VHISS: Venous hemodynamic insufficiency severity score; VM: Valsalva maneuver; VV: Vertebral vein; WM: White matter.

## Competing interest

Robert Zivadinov received personal compensation from Teva Neuroscience, Biogen Idec, EMD Serono, Bayer, Genzyme-Sanofi, Novartis, Claret and General Electric for speaking and consultant fees. He received financial support for research activities from Biogen Idec, Teva Neuroscience, Genzyme-Sanofi, Novartis and EMD Serono.

Chih-Ping Chung has no conflict of interest to report.

## Authors’ contributions

RZ and C-PC conceptualized and designed the study, performed literature research, drafted the manuscript and revised it critically. They approved the final version of the manuscript and both serve as guarantors of the study.
